# Investigation of Some Antiviral *N*-Heterocycles as COVID 19 Drug: Molecular Docking and DFT Calculations

**DOI:** 10.3390/ijms21113922

**Published:** 2020-05-30

**Authors:** Mohamed Hagar, Hoda A. Ahmed, Ghadah Aljohani, Omaima A. Alhaddad

**Affiliations:** 1 Chemistry Department, College of Sciences, Yanbu, Taibah University, Yanbu 30799, Saudi Arabia; 2 Chemistry Department, Faculty of Science, Alexandria University, Alexandria 21321, Egypt; 3 Department of Chemistry, Faculty of Science, Cairo University, Cairo 12613, Egypt; 4 Chemistry Department, College of Sciences, Al-Madina Al-Munawarah, Taibah University, Al-Madina 30002, Saudi Arabia; gjohani@taibahu.edu.sa (G.A.); OHADDAD@taibahu.edu.sa (O.A.A.)

**Keywords:** COVID 19, N-heterocycles, molecular docking, DFT calculations, hydroxychloroquine

## Abstract

The novel coronavirus, COVID-19, caused by SARS-CoV-2, is a global health pandemic that started in December 2019. The effective drug target among coronaviruses is the main protease M^pro^, because of its essential role in processing the polyproteins that are translated from the viral RNA. In this study, the bioactivity of some selected heterocyclic drugs named Favipiravir (**1**), Amodiaquine (**2**), 2′-Fluoro-2′-deoxycytidine (**3**), and Ribavirin (**4**) was evaluated as inhibitors and nucleotide analogues for COVID-19 using computational modeling strategies. The density functional theory (DFT) calculations were performed to estimate the thermal parameters, dipole moment, polarizability, and molecular electrostatic potential of the present drugs; additionally, Mulliken atomic charges of the drugs as well as the chemical reactivity descriptors were investigated. The nominated drugs were docked on SARS-CoV-2 main protease (PDB: 6LU7) to evaluate the binding affinity of these drugs. Besides, the computations data of DFT the docking simulation studies was predicted that the Amodiaquine (**2**) has the least binding energy (−7.77 Kcal/mol) and might serve as a good inhibitor to SARS-CoV-2 comparable with the approved medicines, hydroxychloroquine, and remdesivir which have binding affinity −6.06 and −4.96 Kcal/mol, respectively. The high binding affinity of **2** was attributed to the presence of three hydrogen bonds along with different hydrophobic interactions between the drug and the critical amino acids residues of the receptor. Finally, the estimated molecular electrostatic potential results by DFT were used to illustrate the molecular docking findings. The DFT calculations showed that drug **2** has the highest of lying HOMO, electrophilicity index, basicity, and dipole moment. All these parameters could share with different extent to significantly affect the binding affinity of these drugs with the active protein sites.

## 1. Introduction

Novel coronavirus (COVID-19) emerged as an infection and quickly spread to all countries and is primarily transmitted by contact with infected droplets of saliva or discharge from the nose when infected people cough or sneeze. The first identification of human coronaviruses was observed in the mid-1960s [1,2]. Coronaviruses belong to the family of Coronaviridae, which is a family of enveloped- single stranded- positive sense RNA virus. In addition to, the family of Coronaviridae was divided into four genera: α, β, γ, and δ. Coronaviruses of α and β genera generally infect mammals and humans while the type of γ and δ genera mainly infect birds. That specification is according to the phylogenetic analysis and genome structure of coronaviruses [3]. COVID-19 is a novel coronavirus of the β genus; it is round in shape or crown-shaped and has a diameter of ≈ 60–140 nm, as per observations under the electron microscopy [3]. The incubation period of COVID-19 ranges from 2 to 14 days. According to the World Health Organization, there have been around 1 million and 550 thousand reported cases of COVID-19 disease and more than 90,000 reported deaths to date (11/04/2020). Moreover, SARS-COV-2, the big family of COVID-19, is sensitive to heat and ultraviolet (UV) rays. Furthermore, they are inactivated at 56 °C for half hour and can be stored at −80 °C for many years. Additionally, 75% ethanol, chlorine containing disinfectants and peracetic acid can effectively inactivate COVID-19 [4]. In general, the major principle to prevent and control the infectious disease is eliminating the source of infections thereby protecting the very sensitive population [5]. The hydroxychloroquine an approved drug for malaria disease by FDA was explored as a medication for SARS-CoV-2 [6,7]. Previous reports revealed that, the chloroquine and hydroxychloroquine can inhibit the coronavirus (COVID-19) by changing the pH at the surface of the cell membrane. This action can inhibit the attachment of the virus to the cell membrane. In addition, it can prevent nucleic acid replication, glycosylation of viral proteins, virus assembly, new virus particle delivery, virus release, and other mechanisms to obtain its antiviral effects [8]. Remdesivir, Ebola’s medication [9] demonstrated activity against the coronavirus family in 2017 and 2020 [10,11]. To date, there are no SARS-CoV-2 vaccines available, however several domestic and foreign research institutions and enterprises have used several methods, including mRNA nano-vaccine technology, recombinant or inactivated vaccine technology, and DNA vaccine technology, to develop coronavirus vaccine [5]. The treatment of this virus has not been achieved by any available antiviral agent. Hence, our present work suggests that selection of some derivatives with the appropriate viral restraining mechanisms can offer promising results.

Although the development in the biomedical research has widespread area over the past two decades, the yearly number of new treatments agreed by the U.S. Food and Drug Administration (FDA) has remained relatively limited [12]. Compared to de novo drugs and randomized clinical studies, the drug repurposing for virus including the novel coronavirus is represented as an impacted drug discovery strategy from existing drugs and could essentially shorten the time and reduce the cost [13,14,15,16,17,18]. Recently, Chinese medicinal plants were classified as antiviral inhibitors of the novel COVID 19 [19,20]. The anti-coronavirus effects of these natural derivatives have been confirmed in vitro by direct loading onto cultured cells, however it does not guarantee their effectiveness in vivo. Some protease inhibitors may have an antiviral effect by blocking coronavirus main protease. This assumption was supported by FDA approval about the use of ritonavir/lopinavir, based on data obtained from in vitro studies [21]. So, a recent comparative investigation was established to rationalize the potential use of protease inhibitors as a treatment against infections of SARS-CoV-2 [22].

Computational aspects offer new testable hypotheses for regular drugs involved for novel coronavirus [13,23]. Recently, a virtual screening technique was carried out to identify the active site on the viral protease for the binding of many natural compounds through the molecular docking and cell-based assays [24].

Recently, our research group has focused on the determination of the molecular geometry of synthesized materials via relating the desired properties from experimental evaluation with the estimated parameters from computational calculations [25,26,27].

Heterocycles are widely investigated for possible medicinal applications [25,26,28,29]. Favipiravir (**1**) [30], amodiaquine (**2**) [31], 2′-fluoro-2′-deoxycytidine (**3**) [32], and ribavirin (**4**) [33] are known as antiviral drugs. They inhibit various RNA and DNA viruses. Hence, we aim to determine whether the protease of COVID-19 can be a target protein of these nucleotides in silico using molecular docking. Moreover, a comparative study between these drugs with the FDA approved remdesivir and hydrocloroquine antiviral drugs against a broad range of RNA viruses [11] has been established to investigate the effectiveness of the drugs as inhibitors for COVID 19.

## 2. Results and Discussion

### 2.1. DFT Calculations Studies

**Figure 1:**
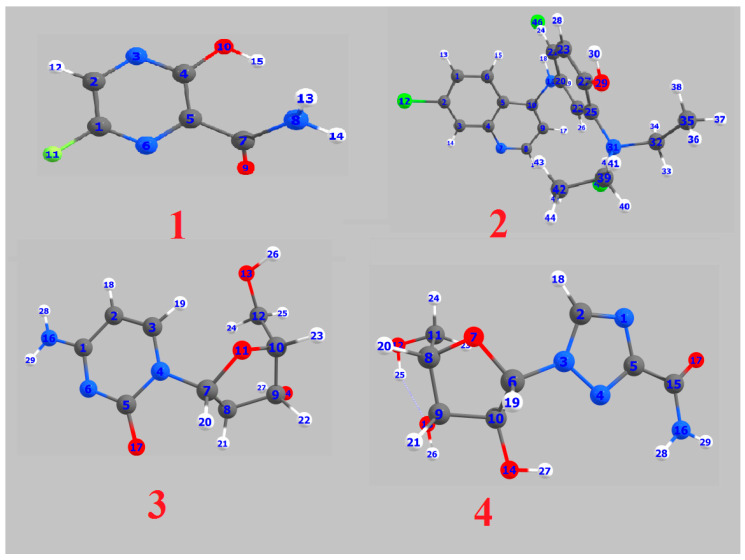
Optimized geometrical structures of present compounds **1–4** with atomic numbering.

The theoretical DFT calculations were performed in gas phase by DFT method at B3LYP 6‒311G (d,p) basis set. All optimum compounds (**1**–**4**, Figure 1) are stable, and this is approved in terms of the absence of the imaginary frequency. The results of the theoretical DFT calculations for all investigated drugs revealed the non-planarity except for compound **1**. The estimated DFT calculations for thermal parameters, dipole moment and the polarizability of the drug derivatives **1–4** are summarized in Table 1.

The DFT estimated data revealed that the dipole moment of the drugs under investigation is in the order of **2** ˃ **4** ˃ **1** ˃ **3**. The high dipole moment **2** and **4** could illustrate their binding pose within a specific target protein and their results of the predicted binding affinity that will be discussed in the following molecular docking part. The polarizability of the materials depends on how the susceptibility of molecular system electron cloud be affected by approaching of a charge. Moreover, it depends on the complexity of the compounds as well as the size of the molecular structure. Molecules of the large size are more polarizable compounds. It is worth noting that the compound **1** is the smallest in size and has the least polarizability (76 Bohr^3^), however, drug **2** of the highest complexity is predicted to have the highest polarizability, 268 Bohr^3^.

#### 2.1.1. Frontier Molecular Orbitals

**Figure 2:**
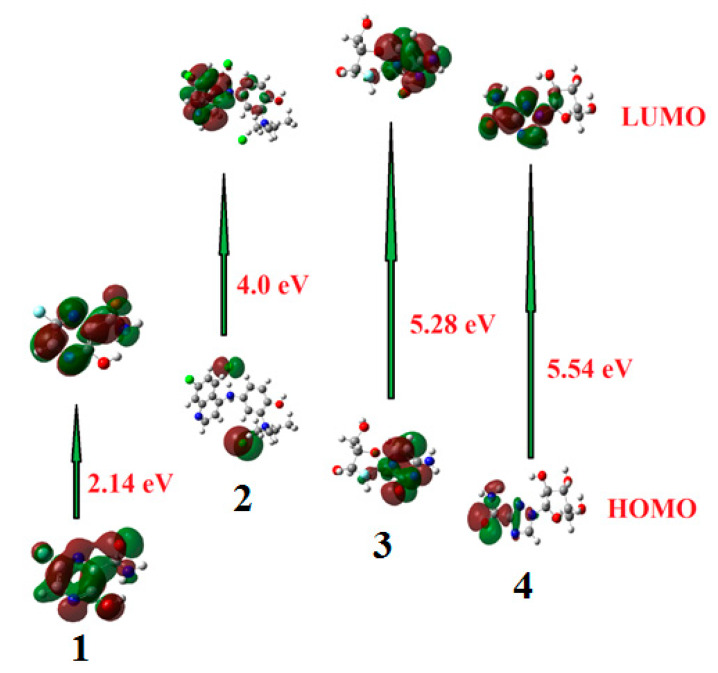
The calculated ground state isodensity surface plots for Frontier molecular orbitals (FMOs) for investigated drugs **1–4**.

Frontier molecular orbitals (FMOs) are the highest occupied molecular orbital (HOMO) and the lowest unoccupied molecular orbital (LUMO). The HOMO is the highest energy orbital occupied with electrons, so it is an electron donor, while, LUMO is the lowest energy orbital that has a space to accept electrons, so it is an electron acceptor. These orbitals control the mode of the interaction of the drugs with other molecules such as the interactions between these drugs and their receptors. The frontier molecular orbitals (FMO) can give realistic qualitative information about susceptibility of the electrons of the HOMO to transfer to the LUMO. Moreover, HOMO and LUMO are very important quantum chemical parameters to determine the reactivity of the molecules and are used to calculate many important parameters such as the chemical reactivity descriptors. The energies of the HOMOs and LUMOs of the studied compounds were calculated using DFT method at B3LYP 6‒311G (d,p) basis set and are tabulated in Table 2. The isodensity surface plots of HOMO and LUMO for investigated compounds are shown in Figure 2.

The results of the FMOs energy analysis revealed that the energies of HOMOs of drugs **1** and **4** are lower compared with the other compounds **2** and **3**. However, the destabilization of the LUMO level is found to be higher in **1** than the others. Consequently, the energy gap of studied drugs is in the order of **1 < 2< 3 < 4**.

Recently, many reports showed that the FMOs have to be taken into consideration in investigation of the structure activity relationships [34,35,36]. The FMOs theory showed that the energy level of the HOMO and the LUMO are the most significant aspects that impact the bioactivities of small structural drugs. Mainly HOMOs that offer electrons, however, the LUMOs accept electrons. Obviously, the level of energy of HOMOs are different for all investigated drugs. Compound **2** showed the most lying HOMO than the other drugs and consequently it could be a better electron donor drug. Interestingly, drug **4** of the largest energy gap **ΔE** = 5.54 eV, there are several hydrophilic interactions that could facilitate the binding with the receptors. This suggests that such hydrophilic interactions considerably impact the binding affinity of such small drugs to the receptors. The HOMO of a certain drug and the LUMO with the adjacent residues could share the orbital interactions during the binding process.

#### 2.1.2. Chemical Reactivity Descriptors

The *E_HOMO_* and *E_LUMO_* are indicators for the prediction of the ionization potential (I= −E_HOMO_) and the electron affinity (A-E_LUMO_) of molecules. Besides the frontier molecular orbitals are used in estimation of other chemical reactivity descriptors such as electronegativity (χ), global hardness (*η*), softness (*δ*), and electrophilicity (*ω*). These are calculated according to the following equations [37]:

(1) $$\chi =-\frac{1}{2}\left(E_{HOMO}+E_{LUMO}\right)$$

(2) $$\eta =-\frac{1}{2}\left(E_{HOMO}-E_{LUMO}\right)$$

(3) $$\delta =\frac{1}{\eta }\;$$

(4) $$\omega =\frac{\chi^{2}}{2\eta }$$

The *χ* value is a prediction of the power of the molecule to attract electrons i.e., Lewis acid, while small values of (χ) are indication of a good base. The global hardness (*η*) is a degree of their charge transfer prohibition, however, the global softness (*δ*) characterizes the ability of a molecule to accept electrons [37]. Soft molecules are of a small energy gap between frontier molecular orbitals and are more reactive than the harder because they could easily transfer electrons to the acceptors.

The electrophilicity (*ω*), calculated from the electronegativity and chemical hardness, is an indicator of lower energy difference due to the highest electron movement between the acceptor, LUMO, and the donor, HOMO.

#### 2.1.3. Molecular Electrostatic Potential (MEP)

To validate the evidence about the reactivity of the drug as inhibitors, the molecular electrostatic potential (MEP) is important to be calculated. Although the MEP gives an indication about the molecular size and shape of the positive, negative as well as the neutral electrostatic potential. These could be a tool to predict physicochemical property relationships with the molecular structure of the drugs under investigation. Moreover, the molecular electrostatic potential is a useful tool to estimate the reactivity of the drugs toward electrophilic and nucleophilic attacks.

**Figure 3:**
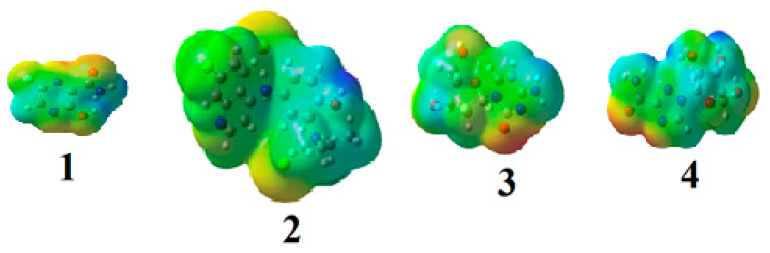
Molecular electrostatic potentials (MEP) of drugs **1–4**.

The molecular electrostatic potential of the studied drugs (**1**–**4**) is calculated by the same method under the same base sets and seen in Figure 3. In the MEP, the maximum negative region is the preferred sites for electrophilic attack, indicated as red color. So, an attacking electrophile will be attracted by the negatively charged sites, and the opposite satiation for the blue regions. It is obvious that the molecular size and the shape as well as the orientation of the negative, positive, and the neutral electrostatic potential varied according to the drug because of the type of the atoms and its electronic nature. The difference in the mapping of the electrostatic potential around the drug could be principally responsible for variation of its binding affinity with the active sites receptor.

#### 2.1.4. Mulliken Atomic Charges

The Mulliken atomic charges of the estimated drugs (**1**–**4**) were calculated the DFT using B3LYP 6 as a method at ‒311G (d,p) at a basis set, the data were tabulated in Table 3. It showed that the C_7_ is the most positive and O_10_ have the most negative charge for drug **1**. On the other hand, it is observed that the most nucleophilic centers of drug **2** are N_11_ and N_31_ which are the most electrophilic susceptibility positions. On the other hand, it is obvious that the nucleophilic susceptibility of the drug **2** is recognized on C_22_ and C_27_ sites. However, N_4_ and N_3_ are the most negative charges of drugs **3** and **4** and their respective positively charged atoms are C_5_ and C_15_**.** The positively charged centers are the most susceptible sites for nucleophilic attacks i.e., electron donation. However, the most negatively charged centers are the most susceptible sites for electrophilic one.

### 2.2. Molecular Docking

**Figure 4:**
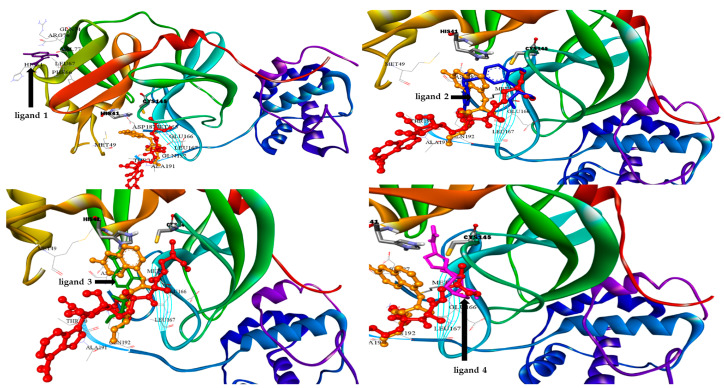
3D docking models of SARS-CoV-2 M^pro^ complexes showing superimposition of the ligand **1** (violet stick), ligand **2** (blue stick), ligand **3** (green stick), ligand **4** (pink stick) with hydroxychloroquine (orange stick and balls) and remdesivir (red stick and balls) in the protein pocket, the amino acids of the catalytic dyad (gray stick).

**Figure 5:**
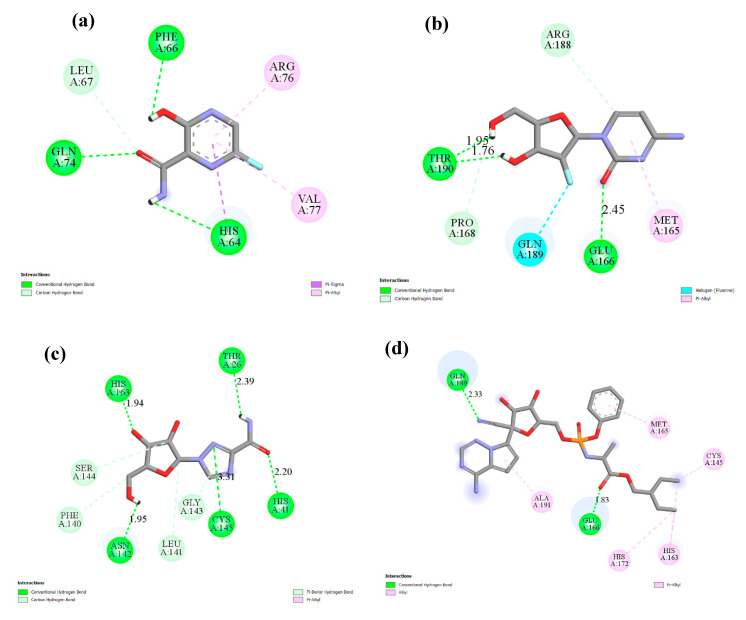
The interacting mode with SARS-CoV-2 M^pro^ in 2D representations in complexes with ligand **1** (**a**), **3** (**b**), **4** (**c**) Remdesivir (**d**).

**Figure 6:**
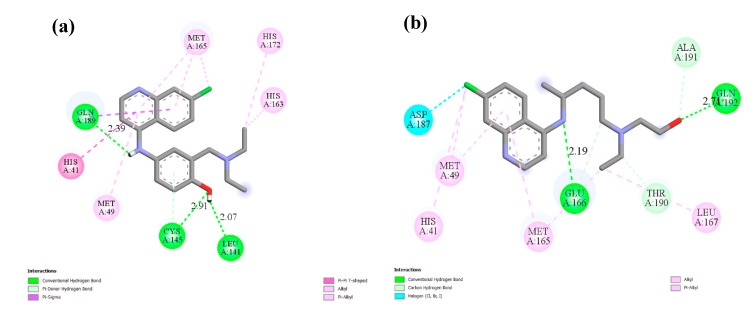
The interacting mode with SARS-CoV-2 M^pro^ in 2D representations of ligand **2** (**a**), hydroxychloroquine (**b**).

Docking simulation studies were done to predict the binding mode of drugs **1**–**4** at the SARS-CoV-2 M^pro^ pocket. Similarly, the hydroxychloroquine and remdesivir were docked in the protein pocket as a control. The binding affinity of compounds **1**–**4** ranged between −4.06 to −7.77 Kcal/mol as presented in Table 4. However, the superpositions of the resulted poses of ligands **1**–**4** with the docking models of hydroxychloroquine and remdesivir, clearly showed that ligand **1** (violet stick) is not located in the catalytic dyad of the receptor (Figure 4). While, ligands **2**–**4** are located in the active pocket similar to hydroxychloroquine and remdesivir. Moreover, ligand **3** (green stick) did not demonstrate any significant interaction with the Cys-His catalytic dyad although it was oriented toward His-41 (Figure 5). Strikingly, ligand **4** is predicted to bind to the catalytic dyad (Cys-145 and His-41) with strong hydrogen bonds along with other residues with binding energy (−4.69 Kcal/mol). The 2D representation of the binding mode of the ligand **4** in the receptor was predicted a high efficiency of the inhibitor with respect to the remdesivir as illustrated in Figure 5. Interestingly, ligand **2** with the least binding energy (−7.77 Kcal/mol) might be better inhibitor to SARS-CoV-2 M^pro^ comparable with the hydroxychloroquine and remdesivir with binding affinity −6.06 and −4.96 Kcal/mol, respectively. Ligand **2** is interacted with the receptor with different hydrophobic interactions besides three hydrogen bonds with amino acids Leu141, Cys145, and Gln189 in a distance 2.07, 2.91, and 2.39 Å (Figure 6). The molecular docking results revealed that the effective interactions of proteins with the drug **2** were found on the atoms (N_11_, N_31_, and O_29_) and this could be attributed to the presence of lone pair electrons on these atoms. Moreover, the π-π stacking could be another hydrophobic interaction between the drug and the receptors.

As previously discussed from the DFT calculations, the electrostatic potential gives an idea about the molecular size and shape of the positive, negative, as well as the neutral electrostatic potential to estimate the reactivity of the studied drugs toward electrophilic and nucleophilic attacks, moreover, van der Waals surface will provide some more negative electrostatic force found on N_11_ (−0.729) N_31_ (−0.742) rather than O_29_ (−0.612) for drug **2**. However, the negative electrostatic potential is localized on N_1_ (−0.336) N_3_ (−0.713) rather than O_7_ (−0.490) for drug **4**. These negative MEP charges could be used for H-bond formation or van der Walls interactions with protein receptors. It is obvious, that the negative electrostatic potential of drug **2** is higher than that of the drug **4**, and this could be an illustration of the least binding affinity of **2** compared with other ligands **1**, **3**, and **4**.

On the other hand, DFT calculations of the frontier molecular orbitals to make a comparison between the energy level of HOMO and LUMO as well as with their energy gap of the investigated drugs showed their impact on the bioactivity of these compounds. The energy levels of HOMOs are between −5.65 eV to −7.61 eV, however, the LUMO are in between −1.05 to 5.47 eV depending on the conjugation as well as the nature of substituents around the nucleus. Further, the order of the FMOs energy gap was 2.14, 4.00, 5.28, and 5.54 for **1**, **2**, **3**, and **4**, respectively. It is clear that, the drug **2** of the high lying HOMO is the most susceptible to be electron donor. Moreover, drugs **2** and **4** are considered good electrophiles because of their high electrophilicity indices 13.69 and 12.80, respectively. Moreover, drug **2** of a high basicity (χ = 3.70) rather than the others could be another effect on the binding affinity [11,38]. Another factor that could affect the degree of the interaction of these drugs with the protein is the dipole moment [39,40,41]. The calculated dipole moments of the investigated drugs are in the range of 14.44–2.26 Debye in the order of **2 > 4 > 1 > 3,** Table 1. All these factors could share together with different extent to significantly impact the degree of the binding affinity of these drugs with the active protein sites.

## 3. Materials and Methods

### 3.1. DFT Calculations

The theoretical estimations were carried out for the investigated drugs by Gaussian 09 software, (2009, Gaussian 09, revision a. 02, gaussian. Inc.: Wallingford, CT, USA) [42]. B3LYP 6–311G basis set was nominated for the DFT calculations. The structural geometry was optimized by minimizing its energies compared to all geometrical variables without forcing any molecular symmetry restrictions. The molecular structure of the optimized drugs has been drawn by Gauss View [43]. The calculated frequency showed that all molecular structures of the investigated drugs was stationary points in the geometry optimization method without the presence of an imaginary frequency.

### 3.2. Molecular Docking Procedure

Docking studies were carried out using the AUTODOCK 4.2 program [44]. The crystal structure of COVID-19 main protease at 2.16 Å resolution was retrieved from the Protein Data Bank (PDB: 6LU7): https://www.rcsb.org/structure/6LU7. The 3D structures in PDB format of hydroxychloroquine (DB01611) and remdesivir (DB14761) were obtained from the DrugBank data base. The selected drugs **1**–**4** were extracted from the PubChem database in the SDF format and were converted to PDB format using PyMOL (The PyMOL Molecular Graphics System, v1.6-alpha; Schrodinger LLC, New York, NY, USA 2013).

Before docking compounds **1**–**4** on the target, the protein was edited using AutoDockTools (ADT). The water molecules were removed, the polar hydrogen atoms were added to the amino acid residues and Gasteiger charges were assigned to all atoms of the protein. Then the protein in PDBQT format was used as an input for the AUTOGRID program. AUTOGRID performed a pre-calculated atomic affinity grid maps for each atom type in the ligand plus an electrostatics map and a separate desolvation map present in the substrate molecule taking the entire protein as the search space. Flexible ligand docking was performed for each compound. Docking calculations were carried out using the Lamarckian genetic algorithm (LGA), and all parameters were the same for each docking. This process was repeated 100 times for each ligand, and the final mean affinity score was taken. The results were shown using Discovery Studio Visualizer v17.2.0.16349 [44].

## 4. Conclusions

Four known antiviral drugs, favipiravir (**1**), amodiaquine (**2**), 2′-fluoro-2′-deoxycytidine (**3**), and ribavirin (**4**), have been investigated as inhibitors for COVID-19 by DFT and molecular docking calculations. The results of the docking studies aimed at studying the binding mode of these drugs to the SARS-CoV 3CLpro. The results revealed that amodiaquine (**2**) and ribavirin (**4**) showed the best affinity with the target receptor, even though this behavior was not experimentally verified. It was predicted that the amodiaquine showed the lowest binding energy (−7.77 Kcal/mol) with respect to the other drugs. Moreover, amodiaquine binding affinity is lower than that of the approved medicines, hydroxychloroquine and remdesivir which have binding affinity −6.06 and −4.96 Kcal/mol; respectively. The results of the molecular docking have been illustrated in terms of the DFT calculations. The DFT results showed that amodiaquine (**2**) is the most lying HOMO, and consequently it could be the best to act as an electron donor. Moreover, the most electrophilic centers of the drug **2** are N_11_, N_31_, and O_29_ and this could be attributed to the presence of the lone pair of electrons on these atoms. Further, the π-π stacking could be another hydrophobic interaction between the drug and the receptors. Additionally, a high basicity (χ = 3.70) and dipole moment (µ = 14.4 Debye) of drug **2** rather than the others, could be the other factors that enhanced the extent of the binding affinity. It could be concluded that these parameters share together with different magnitudes and affect the degree of the binding affinity of these drugs with the active protein sites to afford a certain degree of inhibition. Finally, from these in silico studies for drug **2**, it is very promising to perform further in vitro and small animal model in vivo studies to establish a solid experimental evidence of its activity as inhibitors for COVID-19.

## Figures and Tables

**Table 1:** Thermal parameters (Hartree/Particle), polarizability **α** (Bohr^3^), and dipole moment (Debye) of **1–4**.

Parameter	1	2	3	4
**E_corr_**	0.10	0.38	0.23	0.23
**ZPVE**	−607.34	−2357.00	−914.90	−906.88
**E_tot_**	−607.34	−2356.97	−914.89	−906.86
**H**	−607.33	−2356.97	−914.89	−906.86
**G**	−607.38	−2357.05	−914.94	−906.92
**Total Dipole Moment**	4.32	14.44	2.26	5.94
**Polarizability α**	76.39	268.38	127.41	123.52

ZPVE: Sum of electronic and zero‒point energies; E_tot_: Sum of electronic and thermal energies; H: Sum of electronic and thermal enthalpies; G: Sum of electronic and thermal free energies.

**Table 2:** Calculated electronegativity (**χ**), global hardness (**η**), softness (**δ**), global electrophilicity index (**ω**), the ionization potential (**I**) and the electron affinity (**A**) (in eV) of investigated compounds **1–4.**

Drugs	1	2	3	4
**E_HOMO_**	−7.61	−5.65	−6.34	−7.22
**E_LUMO_**	−5.47	−1.65	−1.05	−1.68
**ΔE**	2.14	4.00	5.28	5.54
**χ**	2.06	3.70	3.65	3.04
**η**	1.07	2.00	2.64	2.77
**δ**	0.94	0.50	0.38	0.36
**ω**	2.27	13.69	17.59	12.80
**I**	7.61	5.65	6.34	7.22
**A**	5.47	1.65	1.05	1.68

**Table 3:** The Mulliken atomic charges of the estimated drugs **1**–**4**.

1	2	3	4
1 C 0.395842	1 C −0.062356	1 C 0.495609	1 N **−0.335624**
2 C −0.045572	2 C −0.295910	2 C −0.095415	2 C 0.510567
3 N −0.238512	3 C 0.034382	3 C 0.456261	**3 N** **−0.512630**
4 C 0.446897	4 C 0.046151	**4 N** **−0.653167**	4 N −0.206644
5 C −0.112549	5 C −0.014008	**5 C 0.557768**	5 C 0.104039
6 N −0.220205	6 C −0.063072	6 N −0.407585	6 C 0.500391
**7 C 0.507551**	7 N −0.272785	7 C 0.428075	7 O **−0.489846**
8 N **−0.831622**	8 C 0.010866	8 C 0.347162	8 C 0.203009
9 O −0.278205	9 C −0.079472	9 C 0.173018	9 C 0.204580
**10 O** **−0.571957**	10 C 0.071445	10 C 0.176333	10 C 0.218767
11 F −0.327241	11 N **−0.729443**	11 O −0.486554	11 C 0.285414
	12 Cl 0.053556	12 C 0.345234	12 O −0.197979
	20 C 0.021858	13 O −0.221785	13 O −0.172743
	21 C −0.034281	14 O −0.210208	14 O −0.219698
	22 C **0.117931**	15 F −0.340011	**15 C 0.585341**
	23 C −0.148508	16 N −0.126724	16 N −0.102687
	25 C 0.030298	17 O −0.438011	17 O −0.374256
	27 C **0.290148**		
	29 O **−0.612097**		
	31 N **−0.742655**		
	32 C −0.283151		
	35 C **−0.516801**		
	39 C −0.296192		
	42 C −0.522817		
	46 Cl −0.904072		
	48 Cl −0.788808		

Bold type: The most positively and negatively charged centers.

**Table 4:** Data analysis of the flexible docking of drugs **1–4,** hydroxychloroquine and remdesivir in the active site of SARS-CoV-2 M^pro^ receptor.

Drug	PubChem CID	Binding Affinity (Kcal/mol)	Amino Acids Residue of SARS-CoV-2 M^pro^
1	492405, favipiravir 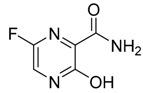	−4.06	His64, Phe66, Leu67, Gln74, Arg76, Val77
2	6246, amodiaquine 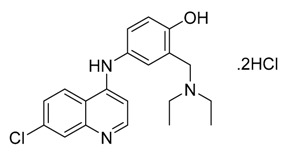	−7.77	His41, Met49, Leu141, Cys145, His163, Met165,His172
3	101507, 2′-fluoro-2′-deoxycytidine 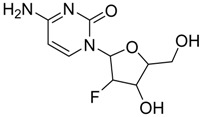	−4.47	Met165, Glu166, Pro168, Arg188, Gln189,Thr190
4	37542, ribavirin 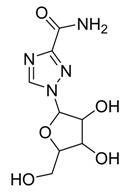	−4.69	Thr26, His41, Phe140, Leu141, Asn142, Gly143, Ser144, Cys145, His163
	Hydroxychloroquine 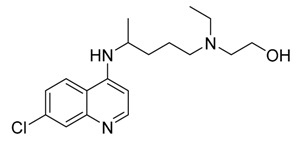	−6.06	His41, Met49, Met165, Glu166, Lue167, Asp187, Ala191, Gln192
	Remdesivir 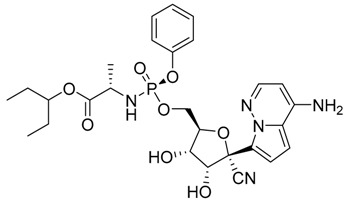	−4.96	Cys145, His163, Met165, Glu166, His172, Gln189, Ala191

Underline: The most interactive amino acid in the binding pocket.
